# *Bacillus cereus* AR156-Induced Resistance to *Colletotrichum acutatum* Is Associated with Priming of Defense Responses in Loquat Fruit

**DOI:** 10.1371/journal.pone.0112494

**Published:** 2014-11-11

**Authors:** Xiaoli Wang, Lei Wang, Jing Wang, Peng Jin, Hongxia Liu, Yonghua Zheng

**Affiliations:** 1 College of Food Science and Technology, Nanjing Agricultural University, Nanjing, Jiangsu, China; 2 College of Life Science and Chemical Engineering, Huaiyin Institute of Technology, Huai'an, Jiangsu, China; 3 College of Plant Protection, Nanjing Agricultural University, Nanjing, Jiangsu, China; South China Agricultural University, China

## Abstract

The effectiveness of a biocontrol agent *Bacillus cereus* AR156 for control of anthracnose rot caused by *Colletotrichum acutatum* in harvested loquat fruit and the possible mechanisms of its action have been investigated. Treatment of fruit with *B. cereus* AR156 resulted in lower disease incidence and smaller lesion diameters compared with that of untreated fruit. The treatment enhanced activities of defense-related enzymes including chitinase, β-1, 3-glucanase, phenylalanine ammonia-lyase, peroxidase and polyphenoloxidase, and promoted accumulation of H_2_O_2_. Total phenolic content and 2,2-diphenyl-1-picrylhydrazyl radical scavenging activity were also increased by treatment. Transcripts of three defense-related genes were enhanced only in fruit undergoing both *B. cereus* AR156 treatment and *C. acutatum* inoculation compared with those receiving either intervention alone. These results suggest that the disease resistance against *C. acutatum* in loquat fruit is enhanced by *B. cereus* AR156 and that the induced resistance is associated with induction and priming of defense responses in the fruit.

## Introduction

Loquat fruit is widely cultivated in the subtropical regions of southern China, Japan, northern India, Israel, and the Mediterranean. The fruit is susceptible to mechanical injury and microbial decay, which limits its storage period and marketing life. Anthracnose rot caused by *Colletotrichum acutatum* is one of the major postharvest diseases of loquat fruit. The disease can be controlled by fungicides, but due to the increasing concerns about the harmful effects of fungicides on the human health and environment, non-chemical disease control methods are becoming important. Among these, the use of biological control appears to be a promising strategy for controlling postharvest diseases of fruit [1].

Some antagonistic yeasts have been reported to effectively inhibit postharvest decay of fruits and vegetables and have shown potential as an alternative to synthetic fungicides [2]. For example, *Pichia membranaefaciens* has been tested as a potential biological control agent for suppressing green mould decay caused by *Penicillium citrinum* on Chinese bayberries [3], anthracnose rot caused by *Colletotrichum acutatum* on loquat fruit [4] and postharvest decay caused by *Monilinia fructicola* on apple fruit [5]. Recently, some strains of *Bacillus* spp. have been evaluated as potential biocontrol agents against postharvest pathogens of peaches [6], [7]. However, the action mechanisms of these biocontrol agents have not been clearly elucidated.

Recently, a rhizobacterium, *Bacillus cereus* AR156, was found to be able to induce disease resistance and inhibit the leaf speck disease caused by *Pseudomonas syringae* pv. *tomato*, the bacterial wilt caused by *Ralstonia solanacearum*, the blight caused by *Phytophthora capsici* Leon., and the root-knot disease caused by *Meloidogyne incognita* in tomato and some other vegetables [8]. These findings indicate that *B. cereus* AR156 may be a promising biocontrol agent against a broad spectrum of pathogens in horticultural crops. The mechanism of *B. cereus* AR156-induced disease resistance was found to be associated with an enhanced capacity to activate defense responses in plant leaves [9], a phenomenon called ‘priming’ [10]. In a more recent work, we demonstrated that *B. cereus* AR156 treatment primed the expression of defense responses in harvested peach fruit, which resulted in enhanced level of induced resistance against *Rhizopus stolonifer* infection [11]. However, whether priming is a common phenomenon of induced resistance in harvested fruits is unknown. The objectives of this study were first to evaluate the efficacy of *B. cereus* AR156 on inducing disease resistance and control of anthracnose rot caused by *C. acutatum* inoculation in harvested loquat fruit, and then to investigate if the *B. cereus* AR156-induced disease resistance against anthracnose rot is associated with induction and priming of defense responses in the fruit.

## Materials and Methods

### Fruit Material

Loquat (*Eriobotrya japonica* Lindl. cv. Jiefangzhong) fruits were hand-harvested at ripe stage from an orchard at Putian of Fujian province, China, and transported within 24 h to our laboratory. We did not conduct any field studies. After arrival, the fruits were selected for uniform size and color and the absence of visual defects. The fruits were then surface-sterilized with 75% ethanol and air dried prior to further treatment.

### Biocontrol Agent and Pathogen

*B. cereus* AR156 was kindly supplied by Prof. Jianhua Guo of College of Plant Protection, Nanjing Agricultural University, China. *B. cereus* AR156 was originally isolated from the forest soil of Zhenjiang City, Jiangsu Province, China [12]. Bacterial cells of *B. cereus* AR156 were grown in Luria-Berta (LB) medium (10 g L^−1^ tryptone, 5 g L^−1^ yeast extract, 10 g L^−1^ NaCl; pH 7.0–7.2) at 28°C with vigorous shaking at 280 rpm for 24 h. Subsequently, bacterial cells were pelleted by centrifugation at 5000 g for 5 min at 20°C in an Avanti-TMJ-25I centrifuge (Beckman, Palo Alto, CA, USA), washed once with, and resuspended in sterile water, and adjusted to 1×10^8^ CFU mL^−1^ for use.

An isolate strain of *C. acutatum*, the challenging pathogen, was obtained from infected loquat fruit and was maintained on potato dextrose agar (PDA) medium (containing the extract of 200 g boiled potatoes, 20 g dextrose and 20 g agar in 1000 mL of distilled water). A conidiospore suspension was prepared from cultures incubated for 14 days at 26°C. Sterile distilled water was used to flood the surface of the PDA culture and conidia were scraped by a sterile loop. The number of spores was calculated with a hemocytometer counting chamber, and then the spore concentration was adjusted to 1×10^5^ spores mL^−1^ with sterile distilled water.

### Efficacy of *B. cereus* AR156 for Control of *C. acutatum*

Two wounds were made at two sides of each loquat fruit with the tip of a sterile dissecting needle and two uniform 4 mm deep and 2 mm wide. 20 µl of washed-cell suspension of *B. cereus* AR156 at 1×10^8^ CFU mL^−1^ was pipetted onto each wound. The wounds were treated with the same amounts of sterile distilled water as the control. Then the fruits were air dried and maintained at 20°C with high humidity (about 95%). After 12 h, each wound was inoculated with 15 µL of a suspension of 1×10^5^ spores of per ml *C. acutatum*. The fruits were then incubated at 20°C with high humidity (about 95%) for 6 days. Each treatment comprised 3 replicates of 30 fruits, and the experiment was conducted twice. Disease incidence (the percentage of wounds showing a decay symptom) and lesion diameter on each fruit wound were observed at 2-day intervals during incubation. Meanwhile, tissue samples were collected from 5 fruits in each replicate. Samples were mixed and immediately frozen in liquid nitrogen, then stored at −80°C until used for enzyme assays and measurements of protein, total phenolic and H_2_O_2_ contents, 2,2-diphenyl-1-picrylhydrazyl (DPPH) radical-scavenging activity.

### Analysis of Defense-related Genes Expression

To further investigate whether *B. cereus* AR156-induced disease resistance against anthracnose rot was associated with priming of defense responses in loquat fruit, tissue samples were collected from 5 fruits before inoculation and at 3, 6 and 12 hours after inoculation to analyze the expression patterns of defense-related genes in loquat fruit only inoculated with distilled water (Mock), *C. acutatum* or *B. cereus* AR156, and in those both treated with *B. cereus* AR156 and inoculated with *C. acutatum*. The non-expressor of pathogenesis-related genes 1 (NPR1) is a central regulator in defense signaling pathways, which leads to increased induction of *PR* genes and enhanced disease resistance [13]. Phenylalanine ammonia-lyase (PAL) is a key enzyme involved in the production of defense-related plant secondary metabolites which are further modified into a wide variety of phenolic compounds [14]. The Ethylene-insensitive 3 (EIN3-like) protein is a transcription factor that participates in the ethylene-dependent defense signaling pathway and play a major role in photosynthetic, developmental, and defense pathways [15]. Therefore, the three defense genes *EjNPR1-like*, *EjPAL2* and *EjEin3-like* were chosen and the housekeeping gene coding for 18S-rRNA was used as a reference in this study.

Total RNA was extracted from loquat fruit according to the method described by Chang et al. [16] with some modifications. RT-PCR was performed using the PrimeScriptTM 16 1st Strand cDNA Synthesis Kit (TaKaRa, Japan). The corresponding nucleotide sequences were obtained from the GENBANK database and used to design gene-specific primer pairs, employing Primer5.0 software. Short and conserved segments of *EjNPR1-like* (GenBank ID: DQ149959), *EjPAL2* (GenBank ID: EF685343) and *EjEin3-like* (GenBank ID: FJ624868) were cloned by degenerate primers. Independent PCR with 25 cycles was performed using aliquots (1 µL) of cDNA samples, and a constitutively expressed gene 18S-rRNA (GenBank ID: AB636342) was used as a quantitative control in the RT-PCR analysis. The sequences of primers used for RT-PCR analysis were as follows:

*EjNPR1-like*_forward: 5′-CGCAA ACCTCAGCAGGACTG-3′,

*EjNPR1-like*_reverse: 5′-TTGTCAACCTCCGGC AAATAC-3′;

*EjPAL2*_forward: 5′-TCTGTCGGGAGGCAGGAATC-3′,

*EjPAL2*_ reverse: 5′- CATCAAAGGATAAGTGGC-3′;

*EjEin3-like*_forward: 5′-GAACAAACTAAGGGAAGGGAAAG-3′,

*EjEin3-like*_ reverse: 5′- CAGCCAGTGGAGGTGGACAT-3′;

18S-rRNA_forward: 5′-AACCTGCACGGCAGAACG-3′,

18S-rRNA_reverse: 5′- GAAGACGACGACGCACCC-3′.

### Assay of Enzyme Activity

Activity of chitinase (EC 3.2.1.14) was measured by the release of N-acetyl-D-glucosamine (NAG) from colloidal chitin according to the method of Abeles et al. [17]. Chitinase was extracted from 1 g of frozen tissue sample with 5 mL of 50 mM sodium acetate buffer (pH 5.0). One unit of chitinase activity is defined as the amount of enzyme required to catalyse the production of 1 µg NAG per hour at 37°C.

β-1,3-Glucanase (EC 3.2.1.58) activity was assayed by measuring the amount of reducing sugar released from the substrate according to the previous method [17]. One g frozen tissue sample was ground with 5 ml of 50 mM sodium acetate buffer (pH 5.0). The crude enzyme extract (1 mL) was incubated for 1 h at 37°C with 1 mL of 4% laminarin (Aldrich, Chemical Co., Milwaukee, WI, USA). The reaction was terminated by heating the sample in boiling water for 5 min and the amount of reducing sugars was measured spectrophotometrically at 540 nm after reaction with 250 µL 3,5-dinitrosalicyclic reagent (Aldrich). One unit is defined as the amount of enzyme catalyzing the formation of 1 µM glucose equivalents in 1 h.

Phenylalanineammonia-lyase (PAL, EC 4.3.1.5) is a key enzyme in the first step of the phenylpropanoid pathway, which is directly involved in the synthesis of phenols, phytoalexins, and lignin that are associated with the localized resistance processes. PAL activity was assayed according to the method of Assis et al. [18] with some modifications. PAL was extracted with 0.2 M sodium borate buffer at pH 8.7 containing 20 mM of β-mercaptoethanol. The assay medium contained 0.1 mL of enzyme extract and 1 mL of l-phenylalanine. After incubation at 40°C for 1 h, the reaction was stopped by adding 0.2 mL of 6 M HCl. One unit of PAL activity is defined as the amount of enzyme that caused an increase of 0.01 in absorbance at 290 nm in 1 h under the assay conditions. The results were expressed as units per mg of protein.

Peroxidase (POD, EC 1.11.1.7) and polyphenoloxidase (PPO, EC 1.10.3.1) are both involved in lignification of host plant cells and considered as key enzymes related to defense reaction against pathogen infections. POD was extracted from 1 g of frozen tissue with 5 ml of 50 mM sodium phosphate buffer (pH 8.7). The extracts were then homogenized and centrifuged at 10,000 g for 20 min at 4°C. POD activity was assayed according to the method of Kochba et al. [19] using guaiacol as donor and H_2_O_2_ as substrate. One unit of POD activity is defined as the amount of enzyme required to cause an increase in absorbance of 0.01 at 470 nm per minute. Enzyme activity was quantified based on the variation of absorbance per minute using the extinction coefficient (26.6 mM^−1^ cm^−1^) according to the following formula:
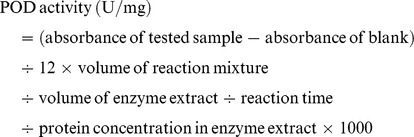



PPO was determined as described by González et al. [20]. Frozen tissue (1 g) was ground with 5 mL of 0.2 M sodium phosphate buffer (pH 6.5), together with 1% of polyvinylpolypyrrolidone. The crude PPO extraction was centrifuged at 10,000 g for 20 min. Each 3 mL of assay medium contained 0.1 M catechol, 0.1 M sodium phosphate buffer (pH 6.5), and 0.1 mL enzyme extract. The increase in absorbance at 420 nm at 25°C was recorded. One unit of PPO activity is defined as the amount of enzyme which caused a change of 1 in absorbance perminute at 420 nm.

Superoxide dismutase (SOD, EC 1.15.1.1) activity was determined by the method of Rao et al. [21] One g of the tissue was ground with 5 mL of 50 mM sodium phosphate buffer (pH 7.8). The reaction mixture contained 50 mM sodium phosphate buffer pH 7.8, 14 mM methionine, 3 µM EDTA, 1 µM nitro blue tetrazolium (NBT), 60 µM riboflavin and 0.1 mL crude enzyme extract. One unit of SOD activity is defined as the amount of enzyme causing 50% inhibition of NBT. The SOD activity was calculated according to the following formula:
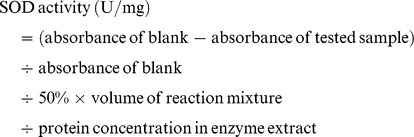



Catalase (CAT, EC 1.11.1.6) activity was performed according to the method of Chance and Maehly [22]. Frozen tissue (1 g) was ground with 5 mL of 50 mM sodium phosphate buffer (pH 7.0). The reaction mixture consisted of 50 mM sodium phosphate buffer (pH 7.0), 12.5 mM H_2_O_2_ and 20 µL of enzyme extract. One unit of CAT activity is defined as the amount of enzyme that decomposed 1 µmol H_2_O_2_ min^−1^ at 30°C. The CAT activity was calculated using the following formula:
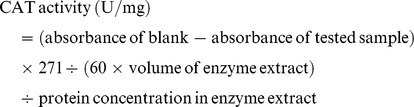



Ascorbate peroxidase (APX, EC 1.11.1.11) activity measurement was adopted from the method of Vicente et al. [23]. One g frozen tissue was ground with 5 mL of 50 mM sodium phosphate buffer (pH 7.0), containing 0.1 mM EDTA, 1 mM ascorbic acid and 1% polyvinyl-pyrrolidone. The homogenate was centrifuged at 10,000 g for 20 min at 4°C and the supernatant was used to determine APX activity. One unit of APX activity is defined as the amount of enzyme that produced an OD290 reduction per minute under the assay conditions.

Protein content in the enzyme extracts was determined by the Bradford [24] method, using bovine serum albumin as a standard. Specific activity of all of the enzymes was expressed as units per milligram of protein.

### Measurement of Total Phenolic, H_2_O_2_ contents and DPPH Radical Scavenging Activity

Total phenolic content was determined using the modified Folin-Ciocalteu procedure described by Slinkard and Singleton [25]. Frozen tissue (1 g) were homogenized in 5 ml of 80% cold acetone and centrifuged at 10,000 g for 20 min; the supernatant was used for analysis. The result was expressed as milligrams of gallic acid equivalent (GAE) per 100 g of fresh weight.

H_2_O_2_ concentrations were determined using a method based on titanium oxidation described by Patterson et al. [26]. Frozen tissue (2 g) was ground and homogenized with 5 mL of chilled 100% acetone and then centrifuged at 10,000 g for 20 min at 4°C. Absorbance of the supernatant was measured at 412 nm. Absorbance values were calibrated against a standard curve (generated using known concentrations of H_2_O_2_) and expressed as µmol g^−1^ FW.

DPPH radical scavenging activity was estimated using the method of Larrauri et al. [27]. Half a gram of frozen sample was extracted with 50% ethanol and centrifuged at 10,000 g for 20 min at 4°C. An ethanolic solution of DPPH served as control. The result was calculated according to the following formula:
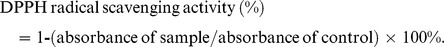



### Statistical Analysis

Experiments were performed using a completely randomized design. All statistical analyses were performed with SPSS 16.0 (SPSS Inc., Chicago, IL, USA) for this study. The data were analyzed by one-way analysis of variance (ANOVA), and mean separations were performed using Duncan's multiple range tests. Significance was defined as *P*<0.05.

## Results

### *B. cereus* AR156 Treatment Controlled Anthracnose Rot in Loquat Fruit

**Figure 1:**
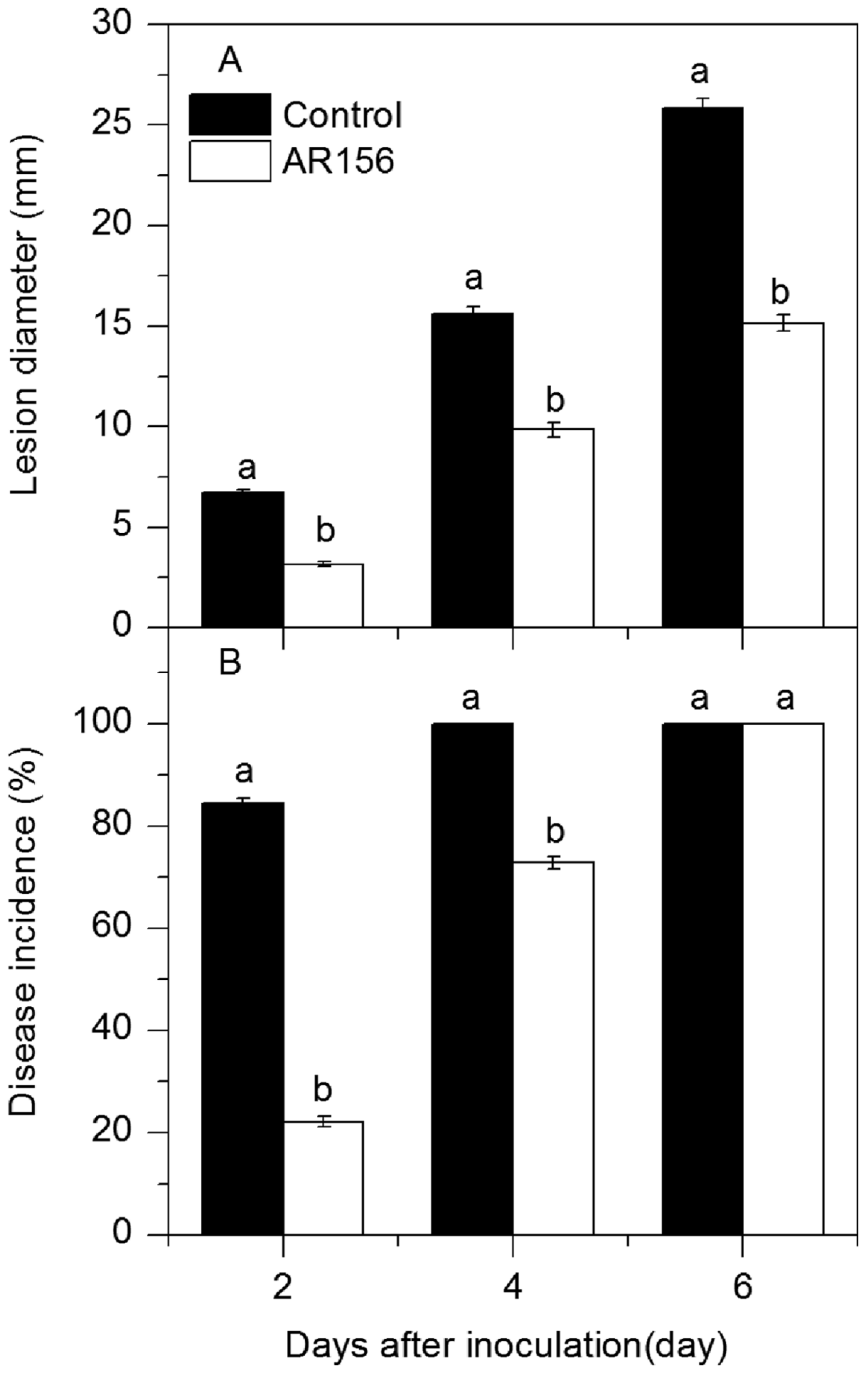
Changes in lesion diameter and disease incidence in loquat fruit. Two wounds were made at two sides of each loquat fruit with the tip of a sterile dissecting needle and two uniform 4 mm deep and 2 mm wide. Twenty µl of washed-cell suspension of *B. cereus* AR156 at 1×10^8^ CFU mL^−1^ was pipetted onto each wound. The wounds that were treated with the same amounts of sterile distilled water served as the control. Then the fruits were air dried and maintained at 20°C with high humidity. After 12 h, each wound was inoculated with 15 µL of a suspension of 1×10^5^ spores of per ml *C. acutatum*. The fruits were then incubated at 20°C with high humidity for 6 days. Changes in lesion diameter (A) and disease incidence (B) in loquat fruit treated with *B. cereus* AR156 and inoculated with *C. acutatum* were observed at 2-day intervals during incubation at 20°C. Each column represents the mean of triplicate samples ± standard errors.

Treatment with *B. cereus* AR156 reduced lesion diameter and disease incidence in loquat fruit inoculated with *C. acutatum* (Figure 1). The lesion diameter in *B. cereus* AR156-treated fruit was 3.2 mm and 9.9 mm on the 2nd and 4th day of incubation at 20°C, which was only 47% and 63%, respectively, of that in control fruit (Figure 1A). Meanwhile, the disease incidence in treated fruit was 26% and 73%, respectively, of that in control fruit on the 2nd and 4th day of incubation (Figure 1B). Although all of the inoculated wounds in both *B. cereus* AR156-treated and control fruit developed decay symptoms after 6 days of inoculation, the lesion diameter in *B. cereus* AR156-treated fruit was lower than that in the control fruit (Figure 1A).

### *B. cereus* AR156 Treatment and *C. acutatum* Inoculation Induced Higher Expression of Defense-related Genes in Loquat Fruit

**Figure 2:**
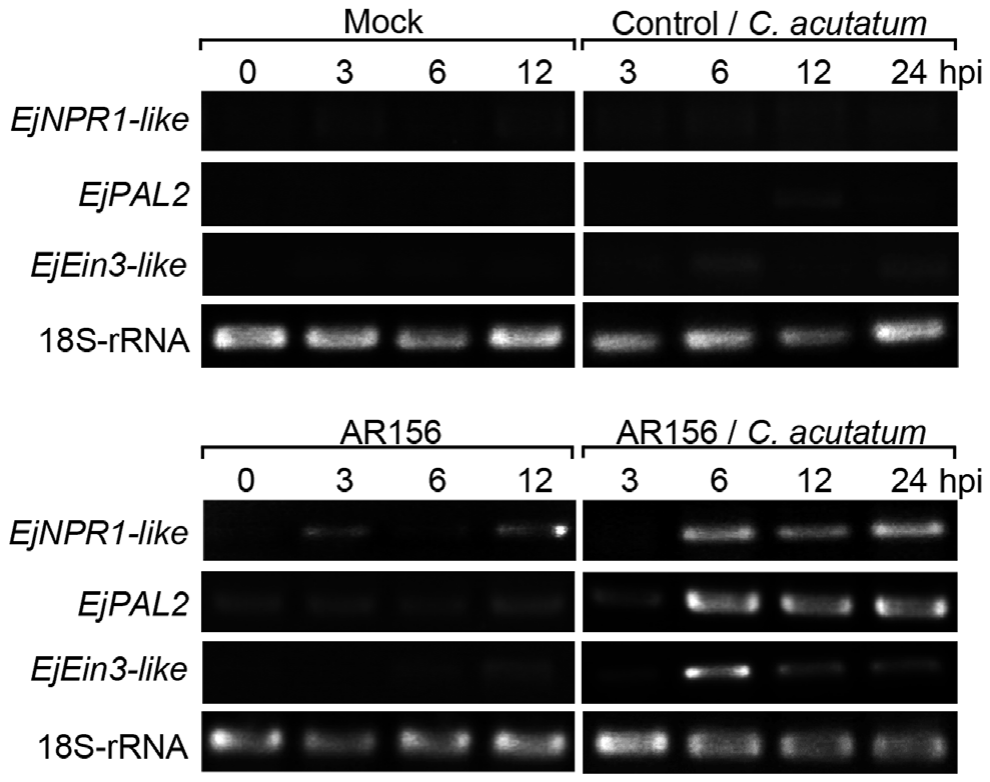
Expression of representative defense-related genes in loquat fruit. Tissue samples were collected from 5 loquat fruits before inoculation with *C. acutatum* and at 3, 6 and 12 hours after inoculation to analyze the expression pattern of *EjNPR1*-*like*, *EjPAL2*, *EjEin3*-*like* genes in loquat fruit only inoculated with distilled water (Mock), *C. acutatum* or *B. cereus* AR156, and in those both treated with *B. cereus* AR156 and inoculated with *C. acutatum*. Reverse transcription-polymerase chain reaction (RT-PCR) was carried out using 18S-rRNA as an internal reference. Each treatment was replicated three times. hpi, hour post inoculation.

Transcripts of *EjNPR1*-*like*, *EjPAL2* and *EjEin3*-*like* genes retained at a very low level in fruit inoculated with *C. acutatum* or distilled water alone (Figure 2). These transcripts were slightly enhanced in fruit treated with *B. cereus* AR156 without inoculation. However, in fruit pre-treated with *B. cereus* AR156 and then inoculated with *C. acutatum*, transcripts of all three tested genes were enhanced and attained higher levels at all the sampling points compared with the other three treatments (Figure 2). Therefore, *B. cereus* AR156 treatment induced higher degree of expression of the three defense-related genes in loquat fruit upon challenge with the pathogen *C. acutatum*.

### *B. cereus* AR156 Treatment Enhanced Chitinase, β-1,3-Glucanase, PAL, POD and PPO Activities in Loquat Fruit

**Figure 3:**
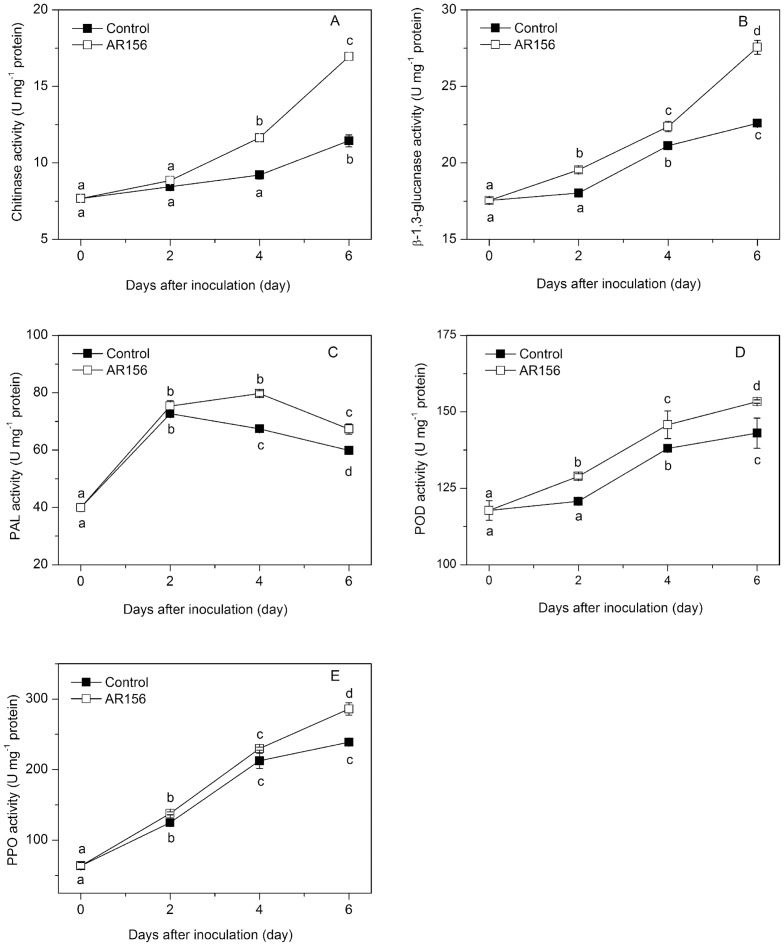
Effect of *B. cereus* AR156 treatment on chitinase, *β*-1,3-glucanase, PAL, POD and PPO activities. Tissue samples were collected from *B. cereus* AR156-treated and untreated loquat fruit before inoculation with *C. acutatum* and at 0, 2, 4 and 6 days after inoculation. The effect of *B. cereus* AR156 treatment on chitinase (A), β-1,3-glucanase (B), PAL (C), POD (D) and PPO (E) activities in loquat fruit during incubation at 20°C was determined. Data are expressed as mean of triplicate samples ± standard errors. Significant differences (*P*<0.05) as indicated by Duncan's multiple range test are shown by different letters.

The activities of chitinase and β-1,3-glucanase in fruit increased gradually during incubation at 20°C (Figure 3A and B). Treatment with *B. cereus* AR156 induced higher activities of chitinase and β-1,3-glucanase during the whole incubation period. The *B. cereus* AR156-treated and *C. acutatum-*inoculated fruit showed 48% higher activity of chitinase and 22% higher activity of β-1,3-glucanase after 6 days of inoculation than the control fruit (Figure 3A and B).

PAL activity in both treated and control fruit increased with incubation time, reaching maximum values on days 2 and 4, respectively, and then decreased during the remainder of incubation. *B. cereus* AR156 treatment promoted the increase and maintained higher PAL activity during the whole storage period compared with control fruit (Figure 3C). Treatment with *B. cereus* AR156 increased POD and PPO activities, both reaching maximum values on day 6 (Figure 3D and E).

### *B. cereus* AR156 Treatment Influenced Activities of SOD, CAT, APX and H_2_O_2_ Content in Loquat Fruit

**Figure 4:**
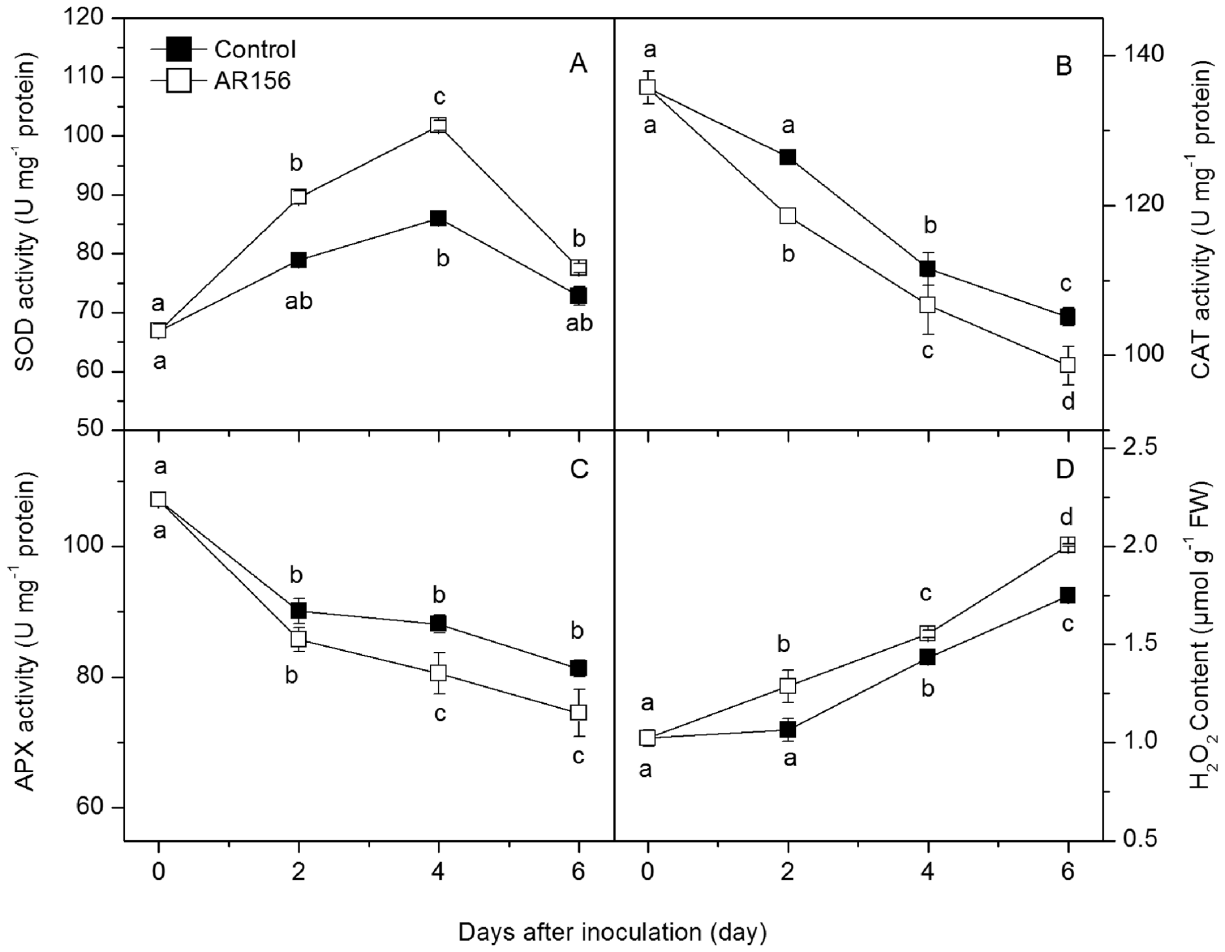
Effect of *B. cereus* AR156 treatment on SOD, CAT, APX activities and H_2_O_2_ content. Tissue samples were collected from *B. cereus* AR156-treated and untreated loquat fruit before inoculation with *C. acutatum* and at 0, 2, 4 and 6 days after inoculation. The effect of *B. cereus* AR156 treatment on SOD (A), CAT (B) and APX (C) activities and H_2_O_2_ content (D) in loquat fruit during incubation at 20°C was determined. Data are expressed as mean of triplicate samples ± standard errors. Significant differences (*P*<0.05) as indicated by Duncan's multiple range test are shown by different letters.

SOD activity in both treated and control fruit increased steadily with incubation time and peaked on day 4 (Figure 4A). *B. cereus* AR156 treatment enhanced the increase in SOD activity during the entire incubation period. The activities of CAT and APX decreased during the incubation. Compared with the control fruit, *B. cereus* AR156 treatment inhibited activities of CAT and APX during the incubation (Figure 4B and C). The level of H_2_O_2_ in both control and *B. cereus* AR156 treated fruit increased during incubation, but higher H_2_O_2_ concentrations were observed in treated fruit throughout the incubation period (Figure 4D).

### *B. cereus* AR156 Treatment Increased Total Phenolic Content and DPPH Radical Scavenging Activity in Loquat Fruit

**Figure 5:**
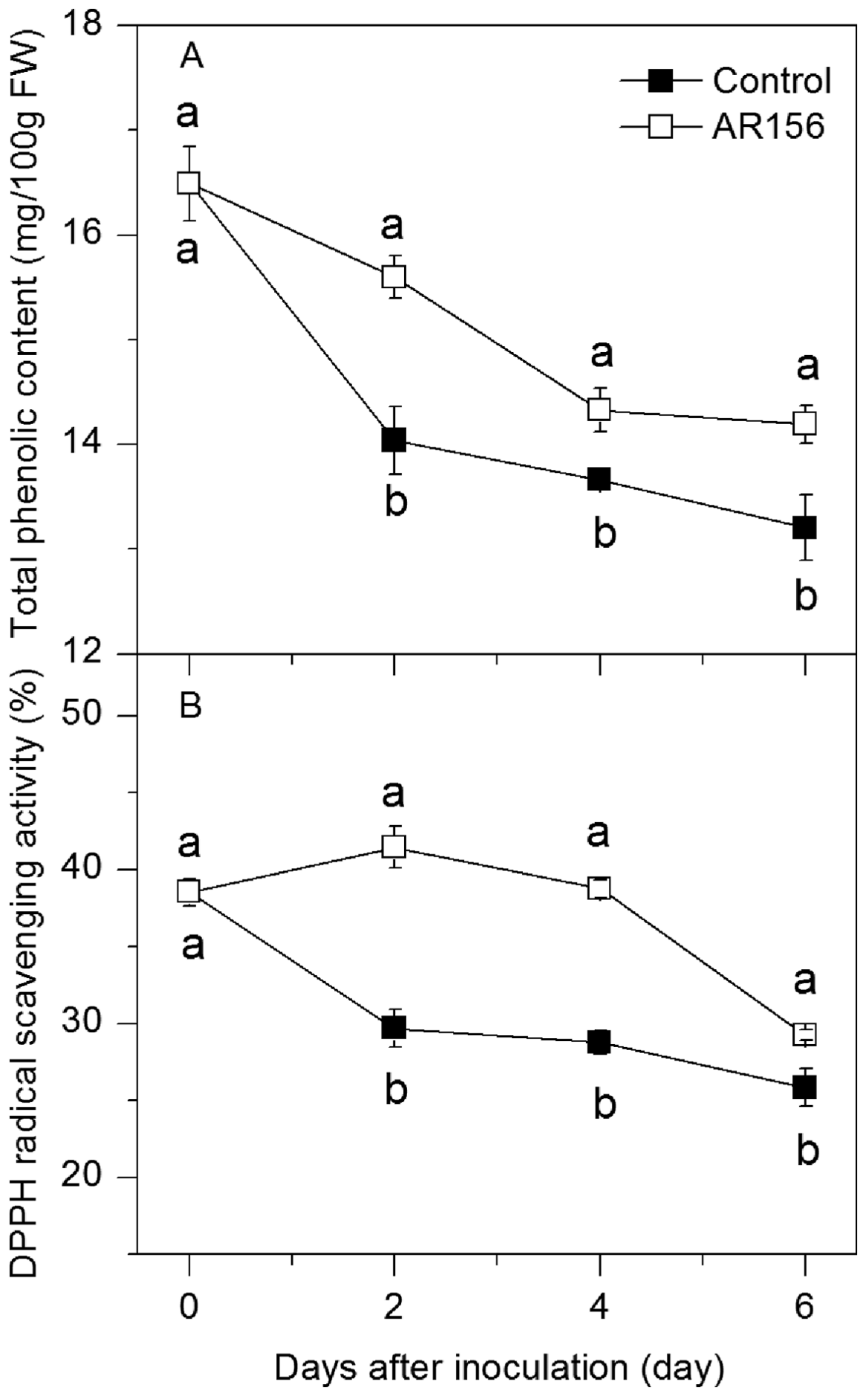
Effect of *B. cereus* AR156 treatment on total phenolic content and DPPH radical scavenging activity. Tissue samples were collected from *B. cereus* AR156-treated and untreated loquat fruit before inoculation with *C. acutatum* and at 0, 2, 4 and 6 days after inoculation. The effect of *B. cereus* AR156 treatment on total phenolic content (A) and DPPH radical scavenging activity (B) in loquat fruit during incubation at 20°C was determined. Data are expressed as mean of triplicate samples ± standard errors. Significant differences (*P*<0.05) as indicated by Duncan's multiple range test are shown by different letters.

The level of total phenolic compounds in both control and *B. cereus* AR156-treated fruit decreased gradually during incubation. Treating the fruit with *B. cereus* AR156 induced the accumulation of total phenolic content, which was higher than that in control fruit during the whole storage period (Figure 5A). DPPH radical scavenging activity in control fruit decreased slightly during storage, while that in *B. cereus* AR156-treated fruit increased slightly and reached a maximum on day 2, and then decreased. Higher DPPH radical scavenging activity was observed in *B. cereus* AR156-treated fruit compared with the control fruit during the entire incubation period (Figure 5B).

## Discussion

*B. cereus* AR156 treatment reduced anthracnose rot incidence and lesion diameter of loquat fruit inoculated with *C. acutatum* (Figure 1). In a previous study [11], we also found that *B. cereus* AR156 treatment suppressed *Rhizopus* rot on peach fruit wounds inoculated with *R. stolonifer*. These results suggest that *B. cereus* AR156 could be a promising biocontrol agent against a broad spectrum of pathogens in harvested fruits.

Although the mechanisms by which microbial antagonists control postharvest diseases have not been clearly elucidated, induced disease resistance has been inferred to be one of the major mode of their actions [2]. Chitinase hydrolyzes the β-1-4-linkage in chitin which is an essential cell wall component of fungi, while β-1,3-glucanase directly degrades cell walls of pathogens or indirectly releases oligosaccharide and elicits defense reactions; therefore both enzymes are thought to be involved in plant defense mechanisms against fungal infection [28]. PAL is the first enzyme in the phenylpropanoid pathway leading to the biosynthesis of phenolics, phytoalexins, lignins and many other compounds associated with localized disease resistance in plants [29], while PPO and POD are both involved in lignification of host plant cells and considered as key enzymes related to defense reaction against pathogen infections [30]. The induction of these defense related enzymes by different biocontrol agents has been observed in harvested apple, Chinese bayberry, loquat and peach fruit, and is correlated to increased disease resistance and reduced disease severity [3]–[5], [11]. In this study, we found that *B. cereus* AR156 induced activities of chitinase, β-1, 3-glucanase, PAL, POD and PPO and suppressed anthracnose rot on loquat fruit wounds inoculated with *C. acutatum* (Figure 1 and 3). These results suggest that the disease resistance of loquat fruit was enhanced by postharvest treatment with *B. cereus* AR156.

Induced resistance protects plants from attacks of a wide spectrum of pathogens. For a long time, it has been assumed that protection by induced resistance was based on the direct activation of defenses by the resistance-inducing agent. However, there is increasing evidence indicating that priming might be a common feature of different types of induced resistance in plants [31]. Shoresh et al. [32] showed that challenge inoculation with the leaf pathogen *Pseudomonas syringae* pv. lachrymans of cucumber plants that had been pre-inoculated with the plant growth-promoting fungus *Trichoderma asperellum* T203 led to augmented pathogenesis-related (PR) gene expression. Verhagen et al. [33] demonstrated that treatment of grapevine plantlets with the well-known induced systemic resistance (ISR) triggering bacteria, *P. fluorescens* CHA0 and *P. aeruginosa* 7NSK2 was effective in protecting grapevine against *Botrytis cinerea* through priming of defense responses. Treatment with the ISR inducing *P. putida* LSW17S did not directly activate the accumulation of *PR* proteins in *tomato* plants, but the plants were primed for enhanced expression of *PR* genes upon infection by *P. syringae* pv. *tomato* DC3000 [34]. To further understand if the *B. cereus* AR156-induced disease resistance against anthracnose rot in this study is associated with priming of defense responses in loquat fruit, we analyzed the expression patterns of three defense-related genes. The results showed that only in fruit that had been pretreated with *B. cereus* AR156 and then challenged with *C. acutatum* had an augmented increase in defense related genes expression observed (Figure 2). This result suggests that the *B. cereus* AR156-induced disease resistance against anthracnose rot in loquat fruit is associated with priming of defense responses, rather than the direct induction. In a previous study [11], we also demonstrated that *B. cereus* AR156 treatment primed the expression of four defense related genes in peach fruit upon challenged with the pathogen of *R. stolonifer*, which resulted in enhanced level of induced resistance and reduced *Rhizopus* rot. These results suggest that priming might be a common phenomenon of *B. cereus* AR156-induced disease resistance in postharvest fruits. Further investigations are needed to elucidate the mechanisms underlying the *B. cereus* AR156-induced priming of defense responses.

Reactive oxygen species (ROS) have the potential to serve not only as protectants against invading pathogens, but also as signals activating further plant defense reactions and can be induced by a variety of biotic or abiotic elicitors [35]. Recently, the role of ROS homeostasis in defense priming has been demonstrated. For example, Zhang et al. [36] showed that rivoflavin-induced resistance against *P. syringae* pv. *tomato* DC3000 was associated with priming of H_2_O_2_ production in *Arabidopsis*. The rivoflavin-induced resistance against *Rhizoctonia solani* in rice was also associated with an augmented accumulation of H_2_O_2_ [37]. Jia et al. [38] demonstrated that treatment with the flavonoid compound quercetin induced resistance against *P. syringae* pv. *tomato* DC3000 by increasing H_2_O_2_ level in *Arabidopsis*. Generally, the metabolism of ROS is controlled by an array of enzymes including SOD, CAT, and APX. Superoxide radical is efficiently converted to H_2_O_2_ by the action of SOD, while H_2_O_2_ is destroyed primarily by APX and CAT. In this study, *B. cereus* AR156 treatment maintained higher SOD activity but lower the activities of CAT and APX, thus resulting in the accumulation of H_2_O_2_. This result suggests that the increased H_2_O_2_ generation may be an important mechanism of *B. cereus* AR156 in priming for enhanced disease resistance in loquat fruit.

In conclusion, our results demonstrate that *B. cereus* AR156 treatment is effective in inducing disease resistance and suppressing anthracnose rot caused by *C. acutatum* in harvested loquat fruit. Moreover, this *B. cereus* AR156-induced disease resistance against *C. acutatum* is associated with priming of H_2_O_2_ production and defense-related genes expression. Defense priming might be a common phenomenon of *B. cereus* AR156-induced disease resistance in harvested fruits.
